# Resolving the time course of visual and auditory object categorization

**DOI:** 10.1152/jn.00515.2021

**Published:** 2022-05-18

**Authors:** Polina Iamshchinina, Agnessa Karapetian, Daniel Kaiser, Radoslaw M. Cichy

**Affiliations:** ^1^Department of Education and Psychology, Freie Universität Berlin, Berlin, Germany; ^2^Berlin School of Mind and Brain, Humboldt-Universität zu Berlin, Berlin, Germany; ^3^Department of Mathematics and Computer Science, Physics, Geography, Mathematical Institute, Justus-Liebig-Universität Gießen, Gießen, Germany; ^4^Center for Mind, Brain and Behavior (CMBB), Philipps-Universität Marburg and Justus-Liebig-Universität Gießen, Marburg, Germany

**Keywords:** auditory modality, EEG, MVPA, object categorization, visual modality

## Abstract

Humans can effortlessly categorize objects, both when they are conveyed through visual images and spoken words. To resolve the neural correlates of object categorization, studies have so far primarily focused on the visual modality. It is therefore still unclear how the brain extracts categorical information from auditory signals. In the current study, we used EEG (*n* = 48) and time-resolved multivariate pattern analysis to investigate *1*) the time course with which object category information emerges in the auditory modality and *2*) how the representational transition from individual object identification to category representation compares between the auditory modality and the visual modality. Our results show that *1*) auditory object category representations can be reliably extracted from EEG signals and *2*) a similar representational transition occurs in the visual and auditory modalities, where an initial representation at the individual-object level is followed by a subsequent representation of the objects’ category membership. Altogether, our results suggest an analogous hierarchy of information processing across sensory channels. However, there was no convergence toward conceptual modality-independent representations, thus providing no evidence for a shared supramodal code.

**NEW & NOTEWORTHY** Object categorization operates on inputs from different sensory modalities, such as vision and audition. This process was mainly studied in vision. Here, we explore auditory object categorization. We show that auditory object category representations can be reliably extracted from EEG signals and, similar to vision, auditory representations initially carry information about individual objects, which is followed by a subsequent representation of the objects’ category membership.

## Graphical abstract

**Figure:**
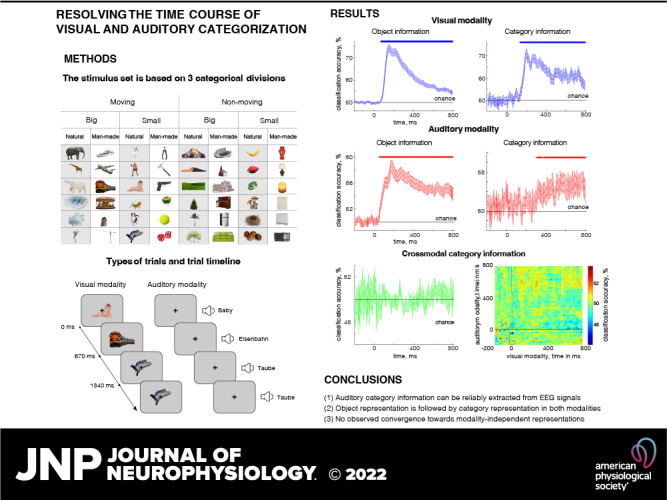

## INTRODUCTION

Whether we see a pineapple or hear somebody say “pineapple,” we can rapidly and effortlessly infer key properties of the object; for instance, we can confidently say that a pineapple is a natural, inanimate object. Such categorization processes are essential for using object knowledge in an efficient way. So far, the studies in the field of object recognition have been investigating the neural correlates of object categorization primarily in the visual modality (1). Using fMRI and M/EEG, researchers have identified a gradual progression from visual representations of individual objects to more abstract representations of an object’s category, both along the ventral visual hierarchy and across processing time (2–8).

By contrast, studies in the field of object recognition seldom focus on object categorization from auditory inputs such as linguistic utterances. Few fMRI studies have pinpointed categorical coding for auditory stimuli to superior temporal and medial frontal cortex (9, 10). Only one EEG study so far has tried to systematically compare the time course of visual and auditory abstract information but did not succeed in reliably establishing category information for auditory stimuli (11). A different line of research investigates the time course of semantic word analysis using event-related potentials. When participants read or listen to full sentences, an N400 ERP component is observed in response to a categorical misattribution of words (12, 13). Yet, the N400 was found to coincide with a wide spectrum of semantic incongruencies (14) and it is currently unclear to what extent the waveform is specific to categorization processes (15).

Here, we pose two critical questions about object recognition from auditory inputs. First, how does object category information dynamically emerge from auditory inputs? Second, is there a representational transition from individual object identification to category membership attribution in the auditory modality and how does it qualitatively compare to the dynamics of object categorization in the visual modality (16)?

To answer these questions, we tracked the emergence of visual and auditory category information in EEG signals. We used a paradigm commonly used in studies of visual object recognition. To evoke automatic category processing and to avoid any context- or task-driven modulations, we presented participants (*n* = 48) with images of objects and spoken words corresponding to the same objects while they were doing an orthogonal one-back task. Objects belonged to three category dimensions, based on object animacy, size, and movement, which were previously shown to explain substantial variance in object representations (17, 18). We used time-resolved multivariate pattern analysis (MVPA) on the resulting EEG data to identify the temporal transition from object-specific to category-defining representations. First, we found that EEG responses after 300 ms of processing form a neural correlate of object categorization in the auditory modality. Second, by tracking representations of individual objects and categories across time, we demonstrate that sensory signals similarly traverse the stages of object identification and categorization in both modalities, suggesting that the perceptual hierarchy established in vision is qualitatively similar to other sensory channels.

## MATERIALS AND METHODS

### Participants

Fifty-one healthy adult participants took part in the study. Three participants had to be excluded due to excessive noise in the data so the final sample consisted of 48 participants (mean age ± SD = 25.02 ± 5.04; 33 female). The study was conducted at the Center for Cognitive Neuroscience Berlin. Participants were compensated with credits or a monetary reward. All participants were native German speakers with normal or corrected-to-normal vision. All participants provided informed written consent. The study was approved by the ethics committee of the Department of Education and Psychology at Freie Universität Berlin.

### Stimuli

The stimulus set was composed of 48 objects each presented as images (2, 11) or as spoken words (9) in German (Fig. 2*A*). The objects were organized according to three orthogonal dimensions, each divided into two categorical divisions: size (big or small), movement (moving or nonmoving), and naturalness [natural or man-made (artificial)]. Each item was assigned to one unique combination of categories along these dimensions (e.g., a baby is small, moving, and natural). The stimulus set was balanced such that each categorical division included one half of the stimulus set (24 objects). The choice of categorical divisions was based on previous studies on visual perception demonstrating that the semantic dimensions spanning these categories yield reliable neural representations independent of experimental design or neuroimaging method (17, 19). The images were selected from Google images using a copyright-free search filter. The size of the images was 400 × 400 pixels. Recordings of the words being spoken were made by the investigators. The words were recorded digitally (at 16 bits with a sampling rate of 44 Hz). They were matched for speaker (same male voice), word length (mean length ± SD = 6.93 ± 2.09 letters: mean number of syllables ± SD = 2.5 ± 0.51, mean duration ± SD = 690 ± 176 ms) but not frequency.

### Experimental Procedure

The experiment was divided into auditory and visual runs. It always started with eight auditory runs, followed by a short break and six visual runs. The auditory runs were always first to prevent participants from imagining the exact same object they had seen during the visual runs and therefore to avoid possible contamination of the results of crossmodal decoding with visual mental imagery during auditory word presentation. We included two more auditory runs than visual runs, as based on pilot data we expected a lower signal-to-noise ratio for auditory signals. Each run consisted of 300 trials and lasted 6 min. Each stimulus was repeated 5 times per run; thus, each stimulus was presented 40 times over the auditory runs and 30 times over the visual runs.

**Figure 1.**
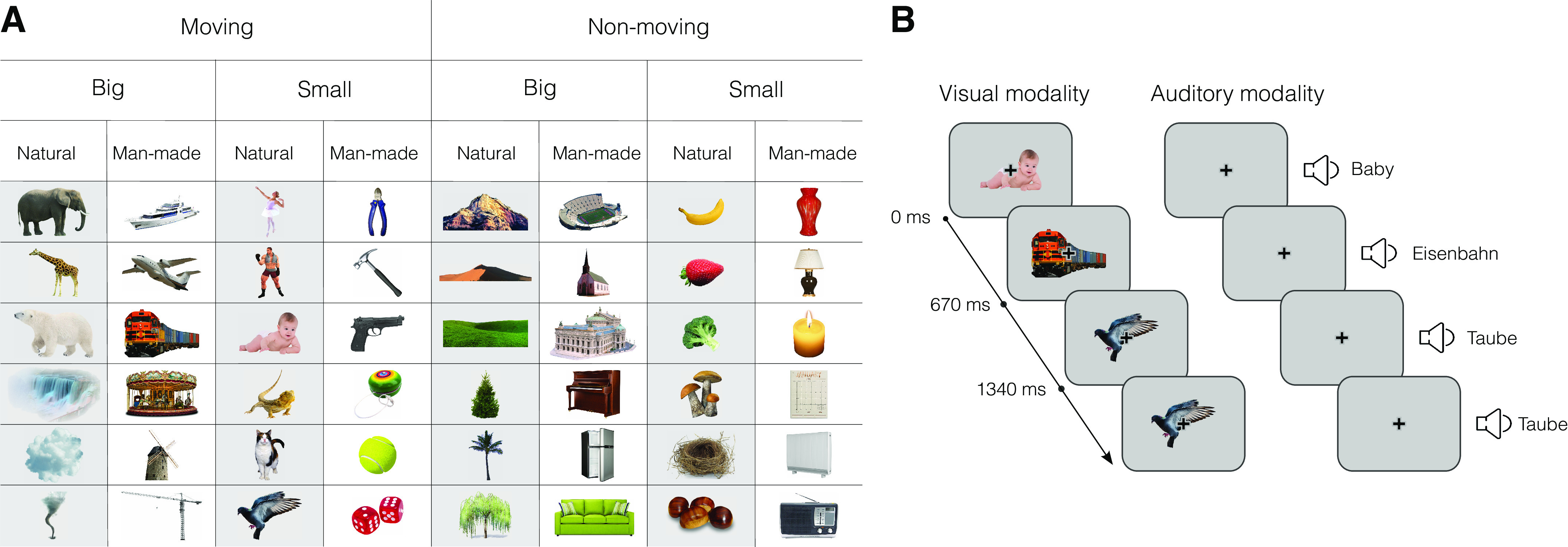
Experimental design. *A*: the stimulus set consisted of 48 objects belonging to 3 categorical divisions. In the visual runs, participants viewed images of these objects, whereas in the auditory runs, they heard the names of the objects. *B*: both in visual (*left*) and auditory (*right*) runs, participants were presented with a random sequence of stimuli. Their task was to press a button when two subsequent stimuli were identical (one-back task).

In visual trials, a pseudorandomly selected stimulus was presented on a gray screen at a visual angle of 4.24°, overlaid with a black fixation cross. In auditory trials, only the fixation cross was present while participants heard the words. For both modalities, stimulus presentation was preceded by a frame with a red fixation cross to aid attention preparation. In 20% of trials, the stimulus was repeated and participants were tasked to press a button (Fig. 1*B*). These one-back repetition trials were excluded from the analysis. To match stimulus durations across modalities, we created a distribution of durations for the visual stimuli based on the duration of the auditory stimuli and randomly assigned these durations to visual stimuli. The intertrial interval (ITI) was jittered (500 ± 50 ms). The ITI after one-back repetition trials was 200 ms longer to allow enough time for a button press. Overall, participants showed good task performance [in auditory runs 93 ± 16% (mean ± SD) correct responses with 390 ± 80 ms reaction time and in visual runs 91 ± 11% correct responses with 450 ± 50 ms reaction time].

### EEG Recording

EEG data were collected using the Easycap 64-electrode system and BrainVision Recorder. The participants wore actiCAP elastic caps, connected to 64 active scalp electrodes: 63 EEG electrodes and 1 reference electrode (Fz). The activity was amplified using the actiCHamp amplifier, sampled at 1,000 Hz, and filtered online between 0.5 and 70 Hz.

### Data Preprocessing

The data were preprocessed offline using the FieldTrip toolbox (20) for MATLAB (release 2018b). The data were first segmented into epochs from 200 ms before stimulus onset to 800 ms poststimulus. Afterward, the data were downsampled to 200 Hz and trials with artifacts were removed (i.e., a trial is excluded if standardized deviations from the mean of all channels in it are larger than 20, for details see jump artifact in fieldtrip toolbox). We performed visual inspection of the data to remove trials that included high-frequency muscle artifacts, spikes across several channels, and eye blink- and head movement-related artifacts (the number of excluded trials never exceeded 10%).

### Classification Analysis

Multivariate pattern analysis (MVPA) was carried out using linear support vector machines (SVMs; libsvm: https://www.csie.ntu.edu.tw/~cjlin/libsvm/) with a fixed cost parameter (c = 1). We performed separate classification analyses on electrode patterns from every millisecond of the epoch across all electrodes. We performed classification on the object and category level, as explained in the following.

For object-level classification, we averaged trials belonging to the same object condition (e.g., ballerina or banana) to increase signal-to-noise ratio (22). In detail, all the trials were first sorted by object condition, i.e., according to the object presented in each particular trial. Within every object condition, the trials were randomly assigned to three distinct trial groups and then averaged within each group, thus forming three “super-trials.” The super-trials were then normalized using multivariate noise normalization (23) to downscale channels with high-noise covariance and thereby improve signal reliability.

The resulting data were used to perform pairwise classification between all possible pairs of objects. Specifically, we trained a classifier with a threefold cross-validation approach using two out of the three super-trials from each of the two object conditions (ballerina vs. banana). We tested the classifier on the left-out super-trial. This classification procedure was repeated 100 times, with different random assignments of trials into the three super-trials. Classification accuracies were averaged across these 100 repetitions. Finally, by averaging all pairwise classification accuracies, we obtained a measure of object-level classification.

For category-level classification, all the trials were sorted according to the object presented in each particular trial and then averaged for each object. Then the object-level averages were sorted by category; this was done three separate times, for each of the three category dimensions (e.g., moving vs. nonmoving, Fig. 1*A*). Within each category division (e.g., moving objects), we randomly assigned the object averages into three groups, and then averaged within each of these groups to form three “super-trials.” Classification was performed in a leave-one-out scheme across the three super-trials as outlined earlier. Critically, the initial averaging of trials at the object level prevented classifiers from training and testing on trials with the same object, thereby probing category-level representations independent of the low-level properties of individual objects in our stimulus set. We again repeated the classification procedure 100 times, with different assignments of the object-level averages into super-trials and averaged the decoding accuracies across these repetitions. Finally, by averaging across all three category distinctions, we obtained a measure of category-level classification.

The comparability between the information time series obtained here and other studies is constrained by the choice of particular stimulus parameters such as the long stimulus duration, nonhomogeneous word frequencies, and the many repetitions per stimulus. For instance, the extensive repetition of individual words may have sped up their disambiguation by anticipating the word meaning before the full word was processed (24).

Here, we repeatedly presented the same exemplar of each object to obtain a higher signal-to-noise ratio per object condition. However, a classifier trained on repeated object exemplars could differentiate low-level features rather than objects limiting the possibility to generalize to category-level information. To address this limitation, we trained a classifier to obtain category-level information on all the objects (trials averaged per object condition) but one (the object condition which was used for testing). In this way, the classifier was designed to generalize across different objects and their features. Future studies could test if similar findings are obtained when multiple exemplars are presented per object condition while the number of repetitions per exemplar is reduced.

### Statistical Analysis

We used nonparametric statistical inference (25), which does not make assumptions about the distribution of the data. Permutation tests were used for cluster-size inference, in which we randomly multiplied the participant-specific data (e.g., EEG decoding accuracies) by +1 or −1 for 10,000 times to create a null distribution. All tests were one-sided against a 50% chance level and thresholded at *P* value < 0.05.

We used a nonparametric test to calculate differences in decoding peak latency between two conditions (object and category information), that is, a difference between the time points at which classification time series reached their maximum accuracy. To estimate if the decoding reaches its peak value in one condition reliably earlier/later than in the other condition, we created 1,000 bootstrapped samples by sampling the participant-specific data with replacement and estimated the peak classification accuracy per each sample. Then, combining the obtained values from all the iterations yielded an empirical distribution of peak latencies in two conditions of interest. Then, we subtracted the peaks estimated in one condition from the peaks estimated in the other condition (object information – category information). We calculated *P* values (one-tail) by dividing the number of bootstrapped samples with differences greater than 0 (e.g., those samples in which the peak latency of object information is later than the peak latency of category information) by the overall number of samples (1,000).

## RESULTS

### The Time Course of Visual Object Representations

Based on previous studies (2–4, 7, 8) revealing a processing hierarchy starting from visual object representations to more abstract category representations, we expected that we could uncover both types of representations from the EEG signals evoked by the object images. Furthermore, we expected that object-level representations would emerge earlier than category representations.

**Figure 2.**
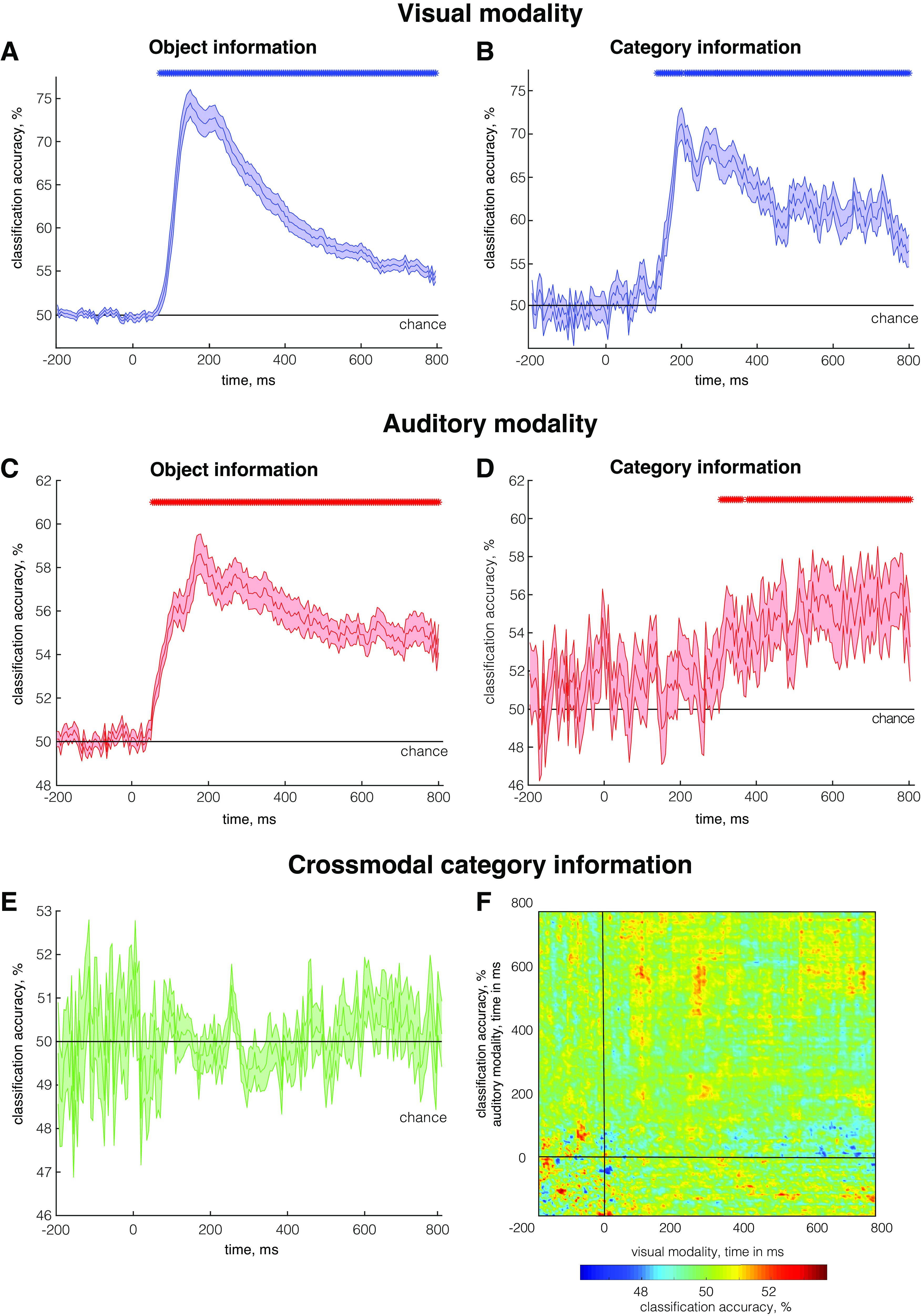
Classification results. *A*: object information time course in the visual modality *B*: category information time course in the visual modality averaged across decoding results obtained for each pair of categorical divisions. *C*: object information time course in the auditory modality *D*: category information time course in the auditory modality averaged across decoding results obtained for each pair of categorical divisions. *E*: category information time course, where classifiers were trained on one modality and tested on the other modality. Results are averaged for both train/test directions. *F*: time generalization results for category information, where classifiers were trained on one modality and tested on the other modality. Results are averaged for both train/test directions. The onset of the stimulus presentation is at 0 ms. Note the different scaling across modalities. Error bars in *A–E* denote between-participant SE. Rows of asterisks in *A–D* indicate significant time points (one-sided permutation test, *P* < 0.05, corrected for multiple comparisons).

We found that EEG signals conveyed significant visual object information from 75 ms to 800 ms after image onset (Fig. 2*A*) and significant category information from 135 ms to 800 ms (Fig. 2*B*). Notably, category information only reached its maximum value significantly after object information (test for peak-to-peak latency difference: *P* = 0.01, see materials and methods), revealing a temporal progression from visual to more abstract representations. Note that given the differences in the two decoding approaches (see materials and methods), absolute decoding accuracies are not directly comparable for the two analyses.

### The Time Course of Auditory Object Representations

Next, we tested whether we could also retrieve object category information when the objects were conveyed through the auditory modality and whether in this case a similar progression from object-level representations to category representations can be observed.

As for the visual modality, we found temporally sustained object information from 55 ms to 800 ms after word onset (Fig. 2*C*). We also found significant category information from 305 ms to 800 ms (Fig. 2*D*), showcasing that object category can be reliably retrieved from auditory brain signals. Furthermore, this category information reached its peak significantly after object-level information (*P* = 0.02), suggesting a similar representational transition toward more abstract, categorical stages of processing in the visual and auditory modalities.

### Commonalities between Visual and Auditory Representations

Finally, we asked whether categorical object representations present in both modalities reflect a convergence toward cconceptual representations that are modality-independent. In case of such a convergence, we should be able to cross classify object category across visual and auditory brain signals. For cross-classification, we trained a classifier on response patterns to each pair of conditions in one modality and tested the classifier on response patterns to the same pairs of conditions from the other modality. In this analysis, no significant cross-decoding was found at any time point across the epoch (Fig. 2*E*).

However, the temporal processing cascades do not necessarily need to match between the visual and auditory modalities. We therefore also performed a time generalization analysis, in which we trained classifiers on each time point in one modality and tested them on all time points in the other modality. Also here, we found no significant cross-decoding. These results indicate that despite the robust category information in both modalities, there is no shared conceptual code for object representation detectable on the level of scalp electrode patterns in our data (Fig. 2*F*).

## DISCUSSION

In this study, we investigated the temporal dynamics of object category processing in the visual and auditory modalities. Specifically, we were interested to know when object category information emerges in the auditory modality and whether the representational transition from object to category level in auditory modality is qualitatively similar to that in vision. Our results show that auditory object category representations can be reliably extracted from EEG signals. Furthermore, they show that there is an analogous representational transition in the visual and auditory modalities, with an initial representation at the individual-object level, and a subsequent representation of the objects’ category membership.

This representational transition has been firmly established in the visual domain before (e.g., 26). Crucially, our study also demonstrates the temporal dynamics of auditory object representations at these different levels of abstraction. Compared with the previous unsuccessful attempt to reveal category information in auditory signals (11), here we used an increased sample size, a greater number of trials per condition, and multivariate noise normalization to improve signal reliability (23). Our results extend previous fMRI research (9, 10) that showed categorical information arising from auditory inputs in superior temporal and medial frontal gyri. These findings suggest that these categorical representations emerge only well after object-level representations, from around 300 ms after the word onset. Notably, in our study, object-level information was temporally sustained together with category information (also Ref. 4). Further research is needed to investigate if this sustained object information is necessary to uphold more abstract representations. The time course of category representation obtained in our study corresponds to the one previously obtained for written words (8), pointing at similarities in processing visual and auditory language information.

The auditory categorical signals in our study temporally align with the occurrence of the N400 component elicited in response to semantically incongruent spoken words (300–900 ms, 27). Several studies specifically demonstrated that the N400 can be evoked by a categorical misattribution of a word (12, 13, 28), thereby hinting at the component as a specific timestamp for word categorization. Building on this research, our findings suggest that extracting the categorical membership during spoken word perception may partially underlie the emergence of N400 in response to categorical misattribution. Further investigation is needed to establish the role of category discrimination in the process of word meaning extraction (27, 29, 30).

Although we found robust category information in both the visual and auditory modalities, we did not find evidence for a transformation of representations from modality-specific codes to modality-independent conceptual representations, as evidenced by the absence of significant crossmodal decoding. In contrast, two fMRI studies identified representations that generalize across the auditory and visual modalities in inferior temporal, inferior frontal, and middle frontal cortices (9, 10). Why did we not find evidence for such representations here? First, crossmodal convergence of representations may be particular to visual and linguistic information being conveyed through the same modality, for instance, for images and written words (9). Second, the current study used an orthogonal task to measure the process of automatic category extraction, which might not sufficiently engage late, modality-independent processes (31, 32). Future studies could employ tasks, such as category verification or story listening/reading (33) that encourage deep processing of words and their context in modality-independent rather than modality-focused fashion. Third, we cannot exclude the possibility that M/EEG scalp sensor patterns lack the sensitivity to uncover the subtle signal differences essential for the readout of modality-unspecific contents (34), while such differences can be revealed with spatially precise fMRI recording (10).

Together, our results elucidate the time course of categorical object coding in the visual and auditory modalities. Furthermore, they establish commonalities in the representational transition from object-level information to categorical representations across the two modalities, suggesting a similarity in the hierarchy of information processing across sensory channels.

## GRANTS

P.I. is supported by the Berlin School of Mind and Brain PhD scholarship. A.K. is supported by a PhD fellowship from Einstein Center for Neurosciences Berlin. D.K. and R.M.C. are supported by Deutsche Forschungsgemeinschaft (DFG) grants (KA4683/2-1, CI241/1-1, CI241/3-1, CI241/7-1). R.M.C. is supported by a European Research Council Starting Grant (ERC-2018-StG).

## DISCLOSURES

No conflicts of interest, financial or otherwise, are declared by the authors.

## AUTHOR CONTRIBUTIONS

P.I., D.K., and R.M.C. conceived and designed research; P.I. and A.K. performed experiments; P.I. and A.K. analyzed data; P.I., A.K., D.K., and R.M.C. interpreted results of experiments; P.I. prepared figures; P.I. drafted manuscript; P.I., A.K., D.K., and R.M.C. edited and revised manuscript; P.I., D.K., and R.M.C. approved final version of manuscript.

## ENDNOTE

At the request of the authors, readers are herein alerted to the fact that additional materials related to this manuscript may be found at https://osf.io/kb35m/?view_only=f4a0fde264554f11a3a12a9109cb72f3 and https://github.com/IamPolina/visual-and-auditory-object-recognition.git. These materials are not a part of this manuscript and have not undergone peer review by the American Physiological Society (APS). APS and the journal editors take no responsibility for these materials, for the website address, or for any links to or from it.

## References

[1] VanRullen R, Thorpe SJ. Is it a bird? Is it a plane? Ultra-rapid visual categorisation of natural and artifactual objects. Perception 30: 655–668, 2001. doi:10.1068/p3029. 11464555

[2] Shinkareva SV, Malave VL, Mason RA, Mitchell TM, Just MA. Commonality of neural representations of words and pictures. Neuroimage 54: 2418–2425, 2011. doi:10.1016/j.neuroimage.2010.10.042. 20974270

[3] Fairhall SL, Caramazza A. Brain regions that represent amodal conceptual knowledge. J Neurosci 33: 10552–10558, 2013. doi:10.1523/JNEUROSCI.0051-13.2013. 23785167 PMC6618586

[4] Cichy RM, Pantazis D, Oliva A. Resolving human object recognition in space and time. Nat Neurosci 17: 455–462, 2014. doi:10.1038/nn.3635. 24464044 PMC4261693

[5] Proklova D, Kaiser D, Peelen MV. Disentangling representations of object shape and object category in human visual cortex: the animate–inanimate distinction. J Cogn Neurosci 28: 680–692, 2016. doi:10.1162/jocn_a_00924. 26765944

[6] Kaiser D, Azzalini DC, Peelen MV. Shape-independent object category responses revealed by MEG and fMRI decoding. J Neurophysiol 115: 2246–2250, 2016. doi:10.1152/jn.01074.2015. 26740535 PMC4869498

[7] Kumar M, Federmeier KD, Fei-Fei L, Beck DM. Evidence for similar patterns of neural activity elicited by picture- and word-based representations of natural scenes. Neuroimage 155: 422–436, 2017. doi:10.1016/j.neuroimage.2017.03.037. 28343000

[8] Leonardelli E, Fait E, Fairhall SL. Temporal dynamics of access to amodal representations of category-level conceptual information. Sci Rep 9: 239, 2019. doi:10.1038/s41598-018-37429-2. 30659237 PMC6338759

[9] Simanova I, Hagoort P, Oostenveld R, van Gerven MAJ. Modality-independent decoding of semantic information from the human brain. Cereb Cortex 24: 426–434, 2014. doi:10.1093/cercor/bhs324. 23064107

[10] Jung Y, Larsen B, Walther DB. Modality-independent coding of scene categories in prefrontal cortex. J Neurosci 38: 5969–5981, 2018. doi:10.1523/JNEUROSCI.0272-18.2018. 29858483 PMC6595974

[11] Simanova I, van Gerven M, Oostenveld R, Hagoort P. Identifying object categories from event-related EEG: toward decoding of conceptual representations. PLoS One 5: e14465, 2010. doi:10.1371/journal.pone.0014465. 21209937 PMC3012689

[12] Fischler I, Childers DG, Achariyapaopan T, Perry NW Jr. Brain potentials during sentence verification: automatic aspects of comprehension. Biol Psychol 21: 83–105, 1985. doi:10.1016/0301-0511(85)90008-0. 4074800

[13] Pulvermüller F, Shtyrov Y, Kujala T, Näätänen R. Word-specific cortical activity as revealed by the mismatch negativity. Psychophysiology 41: 106–112, 2004. doi:10.1111/j.1469-8986.2003.00135.x. 14693005

[14] Kutas M, Federmeier KD. Thirty years and counting: finding meaning in the N400 component of the event-related brain potential (ERP). Annu Rev Psychol 62: 621–647, 2011. doi:10.1146/annurev.psych.093008.131123. 20809790 PMC4052444

[15] Hauk O, Shtyrov Y, Pulvermüller F. The time course of action and action-word comprehension in the human brain as revealed by neurophysiology. J Physiol Paris 102: 50–58, 2008. doi:10.1016/j.jphysparis.2008.03.013. 18485679 PMC2441775

[16] Jiang X, Chevillet MA, Rauschecker JP, Riesenhuber M. Training humans to categorize monkey calls: auditory feature- and category-selective neural tuning changes. Neuron 98: 405–416.e4, 2018. doi:10.1016/j.neuron.2018.03.014. 29673483 PMC7371447

[17] Huth AG, Nishimoto S, Vu AT, Gallant JL. A continuous semantic space describes the representation of thousands of object and action categories across the human brain. Neuron 76: 1210–1224, 2012. doi:10.1016/j.neuron.2012.10.014. 23259955 PMC3556488

[18] Konkle T, Oliva A. A real-world size organization of object responses in occipitotemporal cortex. Neuron 74: 1114–1124, 2012. doi:10.1016/j.neuron.2012.04.036. 22726840 PMC3391318

[19] Konkle T, Caramazza A. Tripartite organization of the ventral stream by animacy and object size. J Neurosci 33: 10235–10242, 2013. doi:10.1523/JNEUROSCI.0983-13.2013. 23785139 PMC3755177

[20] Oostenveld R, Fries P, Maris E, Schoffelen J-M. FieldTrip: Open source software for advanced analysis of MEG, EEG, and invasive electrophysiological data. Comput Intell Neurosci 2011: 156869, 2011. doi:10.1155/2011/156869. 21253357 PMC3021840

[22] Grootswagers T, Wardle SG, Carlson TA. Decoding dynamic brain patterns from evoked responses: a tutorial on multivariate pattern analysis applied to time series neuroimaging data. J Cogn Neurosci 29: 677–697, 2017. doi:10.1162/jocn_a_01068. 27779910

[23] Guggenmos M, Sterzer P, Cichy RM. Multivariate pattern analysis for MEG: a comparison of dissimilarity measures. Neuroimage 173: 434–447, 2018 [Erratum in *Neuroimage* 181: 845, 2018]. doi:10.1016/j.neuroimage.2018.02.044. 29499313

[24] Grosjean F. Spoken word recognition processes and the gating paradigm. Percept Psychophys 28: 267–283, 1980. doi:10.3758/bf03204386. 7465310

[25] Maris E, Oostenveld R. Nonparametric statistical testing of EEG- and MEG-data. J Neurosci Methods 164: 177–190, 2007. doi:10.1016/j.jneumeth.2007.03.024. 17517438

[26] Contini EW, Wardle SG, Carlson TA. Decoding the time-course of object recognition in the human brain: from visual features to categorical decisions. Neuropsychologia 105: 165–176, 2017. doi:10.1016/j.neuropsychologia.2017.02.013. 28215698

[27] Garagnani M, Pulvermüller F. Conceptual grounding of language in action and perception: a neurocomputational model of the emergence of category specificity and semantic hubs. Eur J Neurosci 43: 721–737, 2016. doi:10.1111/ejn.13145. 26660067 PMC4982106

[28] Bentin S, Kutas M, Hillyard SA. Electrophysiological evidence for task effects on semantic priming in auditory word processing. Psychophysiology 30: 161–169, 1993. doi:10.1111/j.1469-8986.1993.tb01729.x. 8434079

[29] Fujihara N, Nageishi Y, Koyama S, Nakajima Y. Electrophysiological evidence for the typicality effect of human cognitive categorization. Int J Psychophysiol 29: 65–75, 1998. doi:10.1016/s0167-8760(97)00099-8. 9641249

[30] Kocagoncu E, Clarke A, Devereux BJ, Tyler LK. Decoding the cortical dynamics of sound-meaning mapping. J Neurosci 37: 1312–1319, 2017. doi:10.1523/JNEUROSCI.2858-16.2016. 28028201 PMC6596862

[31] Tomasello R, Garagnani M, Wennekers T, Pulvermüller F. Brain connections of words, perceptions and actions: A neurobiological model of spatio-temporal semantic activation in the human cortex. Neuropsychologia 98: 111–129, 2017. doi:10.1016/j.neuropsychologia.2016.07.004. 27394150

[32] Meyer GF, Harrison NR, Wuerger SM. The time course of auditory-visual processing of speech and body actions: Evidence for the simultaneous activation of an extended neural network for semantic processing. Neuropsychologia 51: 1716–1725, 2013. doi:10.1016/j.neuropsychologia.2013.05.014. 23727570

[33] Deniz F, Nunez-Elizalde AO, Huth AG, Gallant JL. The representation of semantic information across human cerebral cortex during listening versus reading is invariant to stimulus modality. J Neurosci 39: 7722–7736, 2019. doi:10.1523/JNEUROSCI.0675-19.2019. 31427396 PMC6764208

[34] Giari G, Leonardelli E, Tao Y, Machado M, Fairhall SL. Spatiotemporal properties of the neural representation of conceptual content for words and pictures–an MEG study. Neuroimage 219: 116913, 2020. doi:10.1016/j.neuroimage.2020.116913. 32389730 PMC7343530

